# ASSESSING THE INFLUENCE OF NURSING HOME SIZE AND CARE MODELS ON COVID-19: CASE AND MORTALITY RATES

**DOI:** 10.1093/geroni/igae098.1385

**Published:** 2024-12-31

**Authors:** Yuchi Young, Yichun Liu, Ashley Shayya, Wan-Yu Chiu, Wanhsiang Hsu, Thomas O’Grady

**Affiliations:** State University of New York at Albany, Albany, New York, United States; New York State Department of Health, Albany, New York, United States; University at Albany, SUNY, Albany, New York, United States; National Taiwan University, Taipei, Taipei, Taiwan (Republic of China); University at Albany, SUNY, Albany, New York, United States; University at Albany, SUNY, Albany, New York, United States

## Abstract

The COVID-19 pandemic disproportionately affected older adults in nursing homes, emphasizing the need for comparing various long-term care options. This study compares COVID-19 prevalence and mortality rates in traditional nursing homes, Green Houses (GHs), and nursing homes nested in Continuing Care Retirement Community (CCRC-NHs). CMS data and LTCFocus Data were utilized. COVID-19 case and mortality rates from June 1, 2020, to September 30, 2022, across traditional NHs, GHs, CCRC-NHs in 11 states were included. Poisson regression was utilized to compare risk among types of NHs while adjusting for covariates. The average age of residents of CCRC-NHs and GH was 84.4 and 83.1-years, respectively, higher than those in small (77.5-years) and large (77.6-years) traditional nursing homes (P<.0001). The proportion of female and White residents was significantly higher in CCRC-NHs and GHs than in traditional NH. Compared to GH residents, those in CCRC-NHs (RR 1.50, 95% CI 1.10-2.07), large NHs (RR 1.57, 95% CI 1.14-2.15), and small NHs (RR 1.80, 95% CI 1.32-2.47) had a higher risk of COVID-19 infection, with the highest risk observed in traditional small NHs. There were no statistically significant differences in COVID-19 mortality rates across different types of NHs. This study suggests that nursing home size and care models influence COVID-19 transmission, GHs, with their smaller, person-centered care settings, experienced lower infection rates, highlighting the potential advantages of such environments in pandemic management. Conversely, traditional NHs and CCRC-NH models, with more institutional layouts, may contribute to higher transmission rates suggesting a need for care model reevaluation.

